# TRAFD1 (FLN29) Interacts with Plekhm1 and Regulates Osteoclast Acidification and Resorption

**DOI:** 10.1371/journal.pone.0127537

**Published:** 2015-05-19

**Authors:** Hanna Witwicka, Hong Jia, Artem Kutikov, Pablo Reyes-Gutierrez, Xiangdong Li, Paul R. Odgren

**Affiliations:** Department of Cell and Developmental Biology, University of Massachusetts Medical School, Worcester, Massachusetts, 01655 United States of America; Université de Lyon - Université Jean Monnet, FRANCE

## Abstract

Plekhm1 is a large, multi-modular, adapter protein implicated in osteoclast vesicle trafficking and bone resorption. In patients, inactivating mutations cause osteopetrosis, and gain-of-function mutations cause osteopenia. Investigations of potential Plekhm1 interaction partners by mass spectrometry identified TRAFD1 (FLN29), a protein previously shown to suppress toll-like receptor signaling in monocytes/macrophages, thereby dampening inflammatory responses to innate immunity. We mapped the binding domains to the TRAFD1 zinc finger (aa 37-60), and to the region of Plekhm1 between its second pleckstrin homology domain and its C1 domain (aa 784-986). RANKL slightly increased TRAFD1 levels, particularly in primary osteoclasts, and the co-localization of TRAFD1 with Plekhm1 also increased with RANKL treatment. Stable knockdown of TRAFD1 in RAW 264.7 cells inhibited resorption activity proportionally to the degree of knockdown, and inhibited acidification. The lack of acidification occurred despite the presence of osteoclast acidification factors including carbonic anhydrase II, a3-V-ATPase, and the ClC7 chloride channel. Secretion of TRAP and cathepsin K were also markedly inhibited in knockdown cells. Truncated Plekhm1 in *ia/ia* osteopetrotic rat cells prevented vesicle localization of Plekhm1 and TRAFD1. We conclude that TRAFD1, in association with Plekhm1/Rab7-positive late endosomes-early lysosomes, has a previously unknown role in vesicle trafficking, acidification, and resorption in osteoclasts.

## Introduction

Bone development, remodeling and repair are carried out by osteoblasts which produce bone matrix, osteocytes which communicate the conditions inside the bones, and osteoclasts which resorb bone [1]. Normally the activity of these cells is tightly coupled and regulated. Osteoclasts are multinucleated cells formed by the fusion of mononuclear progenitors of the monocyte/macrophage family that differentiate under the influence of growth factors, macrophage-colony stimulating factor (M-CSF; CSF-1) and the TNF-related cytokine, receptor activator of NF-κB ligand (RANKL), provided in the bone microenvironment by the osteoblasts. A major portion of our current knowledge of osteoclast biology has come from studies of naturally-occurring mutations in human patients and animals with osteopetrosis, as well as from osteopetrosis induced by gene targeting in mice [2–5]. Osteoclasts have developed efficient and unique machinery for dissolving mineral and degrading organic bone matrix [5,6]. When bone resorption is called for, osteoclasts migrate to the site of resorption, attach to the bone, and become highly polarized.

Osteoclast formation from bone marrow precursors is a main point of control in bone resorption, and RANKL and M-CSF are required to induce expression of genes that typify the osteoclast [5]. RANKL binds to its receptor, RANK, on bone marrow progenitor cells and activates TNF receptor associated factors (TRAF proteins). In osteoclasts, only TRAF6 seems to have an essential function. RANK/TRAF6 signaling induces a cascade of signaling events leading to the activation of MAP kinases, NF-κB and AP-1 [7–9]. As a result, these ligands induce NFATc1, the transcription factor considered to be the master regulator of osteoclastogenesis [10].

Membrane dynamics are of central importance in osteoclasts [11–13]. Differentiating osteoclasts develop four distinct and unique membrane domains that are essential for their function, including a sealing zone, ruffled border, facultative secretory domain, and basolateral domain. The first step of polarization involves rearrangement of the actin cytoskeleton and formation of a tight junction between the bone surface and basal membrane to create a ring-like sealing zone. The membrane adjacent to the bone surface within the sealing zone becomes highly convoluted, forming the ruffled border. Through the ruffled border, adjacent to the bone surface, HCl and proteases are released. As a result, bone mineral is dissolved and the proteinaceous matrix is digested. Carbonic anhydrase II (CAII) provides the protons; the vacuolar ATPase (vATPase), including the essential osteoclast-specific a3-subunit, pumps the protons; and the specialized chloride channel ClC7 provides the anions needed for electroneutrality [14]. Loss-of-function mutations in any of those genes cause severe autosomal recessive osteopetrosis (ARO) [4], also known as malignant osteopetrosis. Bone degradation products are then taken up by endocytosis at the ruffled border and further degraded as they are transported by transcytosis to the facultative secretory domain at the top of the polarized osteoclast for secretion [15].

The importance of intracellular vesicle trafficking was emphasized with the discovery of two proteins involved in that process which cause osteoclast bone disease when mutated. One is the sorting nexin, Snx10. Snx10 regulates endosome sorting and movement through the cell. Mutations in *SNX10* account for roughly 4% of ARO in humans, often including an osteopetro-rickets phenotype, and Snx10 is required for osteoclast differentiation and function [16–19]. The other is Plekhm1, a large, multi-domain protein that also causes osteopetrosis in humans and in the incisors absent (*ia*) rat when truncated[20], and which causes osteopenia with focal sclerosis when carrying a gain-of-function point mutation [21]. Plekhm1 associates with late endosomes/early lysosomes, binding to the small GTPase Rab7 and interacting with Rubicon to regulate PI3 kinase-dependent vesicle movement [22]. During our investigations described below, we used mass spectrometry to identify proteins that interact with Plekhm1 in screens of pull-downs of cell extracts. One protein thus identified was TRAFD1 (FLN29).

TRAFD1 was first identified as an interferon- (IFN) and lipopolysaccharide- (LPS) inducible factor [23]. TRAFD1 contains a TRAF-type zinc finger domain at its N-terminus and a TRAF6-binding motif in its middle region. The TRAF6 binding motif of TRAFD1 interacts with the C-terminal part of TRAF6 in monocytes, and TRAFD1 also physically interacts with other TRAF proteins (TRAF1, -2, -3) [24]. In monocytes/macrophages, TRAFD1 suppresses the inflammatory responses to innate immunity by inhibiting Toll-like receptor 4 (TLR4) dependent NF-κB and MAPK activation [23,24]. The interaction with TRAF6 led us to hypothesize that TRAFD1 may be important for osteoclast function.

In the present study, we confirmed and mapped in detail the interaction of TRAFD1 and Plekhm1 We also showed that TRAFD1 expression is up-regulated by RANKL, that it co-localizes with Plekhm1 to late endosomes, and that co-localization of TRAFD1 and Plekhm1 increases after RANKL stimulation. We also show here that TRAFD1 affects osteoclast acidification, mineral solubilization, and secretion of tartrate-resistant acid phosphatase (TRAP) and cathepsin K. In *ia/ia* osteopetrotic rat osteoclasts which are unable to resorb bone, truncated Plekhm1 prevented TRAFD1 association with vesicles.

## Materials and Methods

### Animals

All animals were obtained from our colonies of *ia* rats and C57BL/6J mice maintained at the University of Massachusetts Medical School under specific-pathogen-free conditions, and all procedures were in accordance with the NIH Guide for the Care and Use of Laboratory animals and were approved by the Institutional Animal Care and Use Committee of the University of Massachusetts Medical School. Euthanasia was performed by inhalation anesthesia followed by decapitation.

### Antibodies

The following antibodies were used in the study: rat anti-HA (clone 3F10; Roche Diagnostics, Indianapolis, IN), mouse anti-FLAG M2 (Sigma Aldrich, St. Louis, MO), rabbit anti-lamin B1 (#ab133741; Abcam, Cambridge, MA). Goat anti-Rab7 (# sc-6563), goat anti-ClC7 (# sc-16442), and mouse anti-CAII (# sc-166569) were purchased in Santa Cruz Biotechnology (Dallas, TX). Rabbit anti-Rab7 (# 2094) and rabbit anti-NFAT2 (# 8032) were purchased from Cell Signaling Technology (Danvers, MA). Rabbit anti-cathepsin K was a gift from Dr. Eunice Lee, Shriners Hospital for Children, Montreal, Canada; rabbit anti-TRAP was a gift from Dr. Göran Andersson, Karolinska Institutet, Stockholm, Sweden; rabbit anti-a3-vATPase was a gift from Dr. Beth Lee, Ohio State University, Columbus, OH. Anti-Plekhm1 antibody was raised in rabbits against the N-terminal 497 amino acids of human Plekhm1 protein using a standard immunization protocol (Capralogics Inc., Hardwick, MA), and the antiserum was affinity purified against the immunizing protein using UltraLink Biosupport resin (Thermo Scientific, Rockford, IL). Anti-TRAFD1 antibody was raised in chicken against whole affinity purified human TRAFD1 protein using a standard immunization protocol (Capralogics Inc.). IgY was precipitated from egg yolk and affinity purified with the immunizing antigen using Ultra Link Iodoacetyl Gel resin (Thermo Scientific) according to the manufacturer’s protocol. Secondary HRP-conjugated antibodies used for immunoblotting were: goat anti-rabbit IgG and rabbit anti-goat IgG (Dako, Carpinteria, CA), sheep anti-mouse IgG (GE Healthcare, Piscataway, NJ), and donkey anti-chicken IgY (Gallus Immunotech, Fergus, ON, Canada). Secondary fluorescent dye-conjugated antibodies used for immunofluorescence: donkey anti-chicken IgY, Alexa 488 and TRITC (#703-545-155 and 703-025-155), donkey anti-goat IgG, Alexa 488 (# 705-486-147), and donkey anti-rabbit IgG, Alexa 568 (#711-295-152), purchased from Jackson ImmunoResearch Laboratories Inc. (West Grove, PA). Donkey anti-rabbit IgG, Alexa 488 (#A21206) was from Life Technologies (Woburn, MA).

### Cell cultures

RAW264.7 and HEK 293 cells (ATCC, Manassas, VA) were maintained in α-minimum essential medium (α-MEM) or Dulbecco’s modified Eagle's medium (DMEM), respectively, supplemented with 10% FBS, 1% penicillin/streptomycin in a humidified incubator at 37°C in 5% CO_2_. Bone marrow mononuclear cells (BMMC) and splenocytes were collected from mice (C57BL/6J) and rats (*ia* strain) as described previously [20,25]. These primary mononuclear cells were cultured in α-MEM supplemented with 75 ng/ml of recombinant human M-CSF (Chiron Corp., Emeryville, CA). To generate osteoclasts, RAW264.7 and primary mononuclear cells were cultured for 3–4 days in the presence of 10 ng/ml or 20 ng/ml of soluble mouse RANKL (R&D Systems, Minneapolis, MN), respectively. Medium was changed every other day.

### shRNA gene knockdown and lentivirus gene transfer

TRAFD1-targeting shRNA in pLKO.1 vector was obtained from Sigma Aldrich (SHCLND-NM_006700). shRNA sequences were as follows: shRNA 1: GGCTTGAGTGTGCAGCGTGTTACCTCGAGGTAACACGCTGCACACTCAAGTTTTTG (TRCN0000339582); shRNA2: CGGCATGCCTTACGTTCACTCAATCTCGAGATTGAGTGAACGTAAGGCATGTTTTTG (TRCN0000339580); shRNA3: CGGCACCTACTCGATGTCTCCTAACTCGAGTTAGGAGACATCGAGTAGGTGTTTTTG (TRCN0000339509); shRNA4: CGGCGCACACTTGGACTTCATGTTCTCGAGAACATGAAGTCCAAGTGTGCGTTTTTG (TRCN0000339508). The scrambled shRNA control sequence was: CCTAAGGTTAAGTCGCCCTCGCTCGAGCGAGGGCGACTTAACCTTAGG, generated by Sarbassov et al. [26] and obtained from Addgene, Cambridge, MA (plasmid # 1864). shRNA constructs in the pLKO.1 vector were packaged into lentivirus by transfection into HEK293 cells together with packaging vector pCMV d8.2 dvpr (plasmid # 8455, Addgene) and envelope vector pCMV-VSV-G (plasmid # 8454, Addgene). Transfections were carried out using Fugene 6 (Roche Diagnostics). Viral supernatant was collected at 48 h post-transfection. Viral infections of RAW264.7 cells were carried out at a multiplicity of infection of 1 with 8 μg/ml of hexadimethrine bromide. To obtain stable cell lines with TRAFD1 knocked down, colonies of virally transduced RAW264.7 cells were selected by culturing in medium containing 3 μg/ml of puromycin (Sigma Aldrich). Cells were routinely maintained in 1 μg/ml puromycin. Knock down efficiency was confirmed using RT-qPCR with gene expression normalized to acidic ribosomal phosphoprotein P0 (*Rplp0*) [27].

### Tandem-affinity purification and mass spectrometry

The pIRES-puro Glue vector (TAP tagged), generated by Angers et al., [28], was purchased from Addgene (plasmid # 15100). Full-length cDNA for human Plekhm1 was amplified and cloned into the pIRES-puro Glue vector downstream of the dual affinity tag. Construct sequences were verified by sequencing. The purification procedure was described previously [28]. Briefly, cell lysate from HEK293 cells (5 × 10^8^) stably expressing low levels of TAP-tagged Plekhm1 was incubated overnight at 4°C with 100 μl packed volume of Streptavidin Sepharose (GE Healthcare). Streptavidin beads were washed and protein complexes were eluted from the streptavidin resin in calmodulin binding buffer supplemented with 50 mM biotin. The second round of affinity purification was performed using 100 μl of Calmodulin Sepharose (GE Healthcare). After washing, the protein complexes were eluted twice with 100 μl wash buffer containing EGTA. The eluates were combined, run briefly into an SDS-PAGE gel, excised, and sent to the Proteomic and Mass Spectrometry Facility at University of Massachusetts Medical School to be digested with trypsin and analyzed by LC-MS/MS.

### Immunoprecipitation/pull down

Full-length human cDNA for Plekhm1 and various deletion mutants were PCR-amplified and cloned into the pIRES-puro Glue vector downstream of the tandem affinity tags. The p3×FLAG-CMV14 plasmid with human cDNA for TRAFD1 was a gift from Dr. Akihiko Yoshimura, Keio University, Tokyo, Japan). Deletion mutants of the cDNA were PCR-amplified and cloned into the p3×FLAG-CMV14. A 6 cm dish of HEK293T cells was transfected with p3xFLAG-CMV14-TRAFD1 and pIRES-puro Glue–Plekhm1, either full-length or deletion mutants using Lipofectamine 2000 (LifeTechnologies). 24 hours post-transfection, the cells were washed with ice-cold PBS twice and lysed in 0.5 ml of lysis buffer (50 mM Hepes-KOH pH 8.0, 100 mM KCl, 2 mM EDTA, 0.1% Nonidet-40, 10% glycerol, 10 mM NaF, 0.5 mM Na_3_VO_4_) supplemented with fresh 2 mM DTT and protease inhibitor cocktail (Sigma Aldrich). Lysates were cleared by centrifugation and pull-downs were performed using 25 μl of Streptavidin Sepharose for 2 hours at 4°C. After extensive washing of beads with lysis buffer, precipitated proteins were detected by immunoblot analysis using chemiluminescent detection. For immunoprecipitation of FLAG-tagged TRAFD1, HEK293 cells were lysed with lysis buffer (50 mM Tris HCl pH 7.4, 150 mM NaCl, 5 mM MgCl_2_, 0.5% Triton-X-100 supplemented with protease inhibitor cocktail). 15 μl of FLAG-M2 gel (Sigma Aldrich) was incubated with cell lysate for 2 hours at 4 C and pellets were washed 5 times with wash buffer (20 mM Tris, pH7.4, 150 mM NaCl, 0.2% Triton X-100, 2.5 mM MgCl_2_) prior to loading onto SDS-PAGE gels for analysis.

### Immunofluorescence

Cells grown on glass coverslips were fixed with 3% paraformaldehyde in PBS for 10 min, washed with PBS, permeabilized with 1% Triton X-100, and blocked with 1% BSA with 0.05% Tween 20 in PBS. Primary and secondary antibodies were diluted in blocking solution. Primary antibodies were used as follows: chicken anti-TRAFD1 (1:25), rabbit anti-Plekhm1 (1:1000), goat anti-Rab7 (1:100). All were incubated for 1 hour at room temperature and rinsed. Fluorescent dye-conjugated secondary antibodies were then incubated for 45 minutes. DAPI (Life Technologies) staining was used to label nuclei. The samples were mounted with ProLong Gold antifade reagent (Life Technologies) and observed with a Leica SP5 (II) laser scanning confocal microscope (Leica, Buffalo Grove, IL) equipped with 40 × (1.30 NA) and 63 × (1.4–0.6 NA) oil immersion lenses. Leica LAS AF Lite software was used for recording and image processing.

### RNA isolation and quantitative RT-PCR

RNA was extracted using RNeasy (Qiagen, Germantown, MD) and the yield determined by measuring OD_260_. 1 μg of total RNA was subjected to reverse transcription with a QuantiTect Reverse Transcription Kit (Qiagen). The resulting cDNA was used for PCR using a QuantiFast SYBR Green PCR kit (Qiagen). Amplification reactions were performed in triplicate in 10 μl final volume that included the following: 10–50 ng of template, 1 μM primers, 2× SYBR Green Master Mix. Reactions were processed in a LightCycler 2 (Roche Diagnostics): 95°C for 5 min, then 40 cycles of 95°C for 10 s and 60°C for 30 s. Δ*C*_t_ for each gene was calculated and represents the difference between the *C*_t_ value for the gene of interest and that of the reference gene. Fold-changes were calculated using the 2^*−*ΔΔ*C*^_t_ convention [29]. Primer sequences and the accession numbers of cDNA targets are shown in S1 Table (supplementary materials).

### Resorption assay

Cells at a density 15,000/well were cultured for 10 days on Osteo Assay Surface (hydroxyapatite) 24-well plates (Corning, Tewksbury, MA) under differentiation conditions. To observe resorption pits, plates were gently stripped of cells with 10% bleach, rinsed with distilled water, air dried, and scanned on a flatbed scanner (Microtek 9800 XL). The percentage of resorbed area was analyzed by NIH ImageJ software.

### TRAP staining

Fixed cells were TRAP-stained using a leukocyte acid phosphatase kit (Sigma Aldrich) according to the manufacturer’s instructions. TRAP-positive multinucleated cells containing three or more nuclei were counted.

### TRAP activity assay

RAW264.7 cells were cultured in α-MEM containing RANKL on HA plates until differentiated (3–4 days). On the day when cells differentiated, the medium was changed. Conditioned medium was collected on subsequent days 2 and 5 for measurement of TRAP activity as described previously [30]. Absorbance was measured at 405nm. TRAP activity was calculated as the difference between wells with and without sodium molybdate.

### Cathepsin K secretion assay

RAW264.7 cells were differentiated on HA plates, as above. When cells differentiated, the medium was changed, and at the time points indicated, conditioned medium was collected. Cells from each well were also collected and total cell protein extracts were prepared by lysis in RIPA buffer. Supernatant (40 ul) and total cell lysate (50 ug) were resolved on SDS-PAGE gels, blotted onto PVDF, and probed with anti-cathespin K. Blots were subsequently probed with anti-lamin B1 antibody as a loading control.

### Acidification assay

RAW264.7 cells were cultured for 5 days on Osteo Assay 24-well plates under differentiation conditions. Acridine orange (3,6-bis[Dimethyloamine] acridine] at 10 μg/ml was loaded for 45 minutes in the culture medium. The dye was rinsed out and the cells were observed by inverted fluorescence microscopy (Leica DMI6000) [31]. As a control, some cells were treated with the proton pump inhibitor bafilomycin A (200 nM; Enzo Life Science, Farmingdale, NY).

### Subcellular fractionation

Mononuclear cells isolated from wild-type (WT) and *ia/ia* rats were grown in 150 mm dishes in the presence of RANKL (20 ng/ml) until differentiated (4 days). The cell fractionation protocol was adapted from [32] with modifications. Briefly, cells (1.7× 10^6^) were washed twice with PBS and scraped into hypo-osmotic buffer (150 mM sucrose; 10 mM Tris; 1 mM EDTA, pH 7.5 supplemented with protease inhibitor cocktail). After 5 minutes on ice, cells were homogenized by 10 passes through a 23Ga needle attached to a 1 ml syringe and diluted with an equal volume of hyper-osmotic buffer (the same as above, but with 450 mM sucrose). The cell lysate was then centrifuged at 1000 G_av_ for 10 minutes at 4°C. The nuclear pellet from this centrifugation was resuspended in RIPA buffer (50 mM Tris-HCl, pH 7.4; 150 mM NaCl; 1% NP-40; 0.5% Na-deoxycholate; 0.1% SDS; 2 mM EDTA; 10 mM NaF). The post-nuclear supernatant was collected into a new tube and centrifuged at 15,000 G_av_ for 15 minutes at 4°C. The pelleted light mitochondrial fraction (LMF) was resuspended in RIPA buffer and the post-mitochondrial supernatant was further centrifuged at 100,000 G_av_ for 60 minutes at 4°C. The pelleted vesicle fraction was resuspended in RIPA buffer and the remaining supernatant was saved as the cytosolic fraction. Protein fractions were frozen at -80°C until analyzed. Nuclear and cytosolic cell extracts were isolated according Kloet et al. [33].

### Western blotting

Protein concentrations of whole cell extracts were determined using Coomassie Plus Protein Assay Reagent (Thermo Scientific) according to the manufacturer’s instructions. 25–150 μg of total protein was loaded per lane for SDS-PAGE. The gels were blotted onto PVDF membranes and probed with the following primary antibodies at the indicated dilutions: 1:10,000 Plekhm1; 1:250 TRAFD1; 1:1000 Rab7 (Cell Signaling); 1:1000 CAII, 1:2000 cathepsin K; 1:2000 TRAP; 1:3000 a3-vATPase; 1:1000 HA; 1:5000 FLAG M2; 1:500 α-tubulin; and 1:5000 lamin B1. The appropriate horseradish peroxidase-conjugated secondary antibody was used at 1:2000 to 1:5000 dilution. Proteins of interest were visualized with ECL substrate either by film exposure (Thermo Scientific) or Gel Doc XR^+^ Imaging System (Bio Rad).

### Statistical analysis

All quantitative data are shown as mean + s.d. of at least three (n = 3) biological replicates for each experiment. Two-tailed Student's *t*-test (Microsoft Excel or GraphPad Prism 6) was used for statistical comparisons of two groups, while one-way ANOVA (GraphPad Prism 6) was used to compare more than two groups. For all analyses a *P*-value of less than 0.05 was considered to be statistically significant.

### Co-localization analysis

Co-localization analysis was done using Just Another Co-localisation Plugin (JACoP) for Image J (NIH), in which Pearson’s correlation coefficient values were analyzed. A value of 1 represents perfect correlation, while a value close to 0 represents random correlation. To eliminate the possibility of false-positives, one of the images was rotated 90° and co-localization was measured again **(S1 Fig).** Values obtained were close to zero, validating the positive co-localizations in the non-rotated images [34].

## Results

### Identification of TRAFD1 as a Plekhm1 binding protein

**Fig 1:**
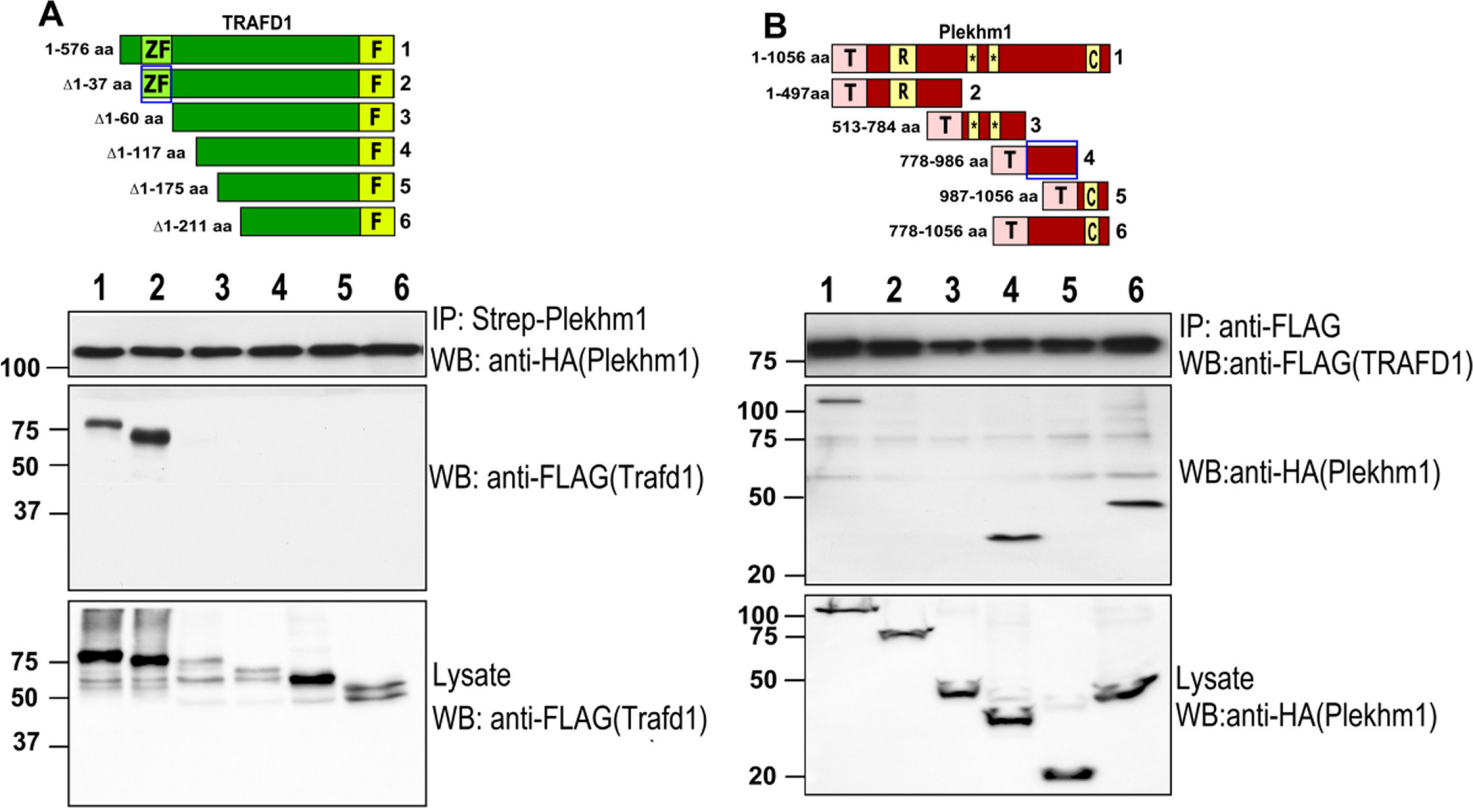
Binding of Plekhm1 with TRAFD1. (A) Pull-down of full-length, N-terminally TAP-tagged (streptavidin binding protein:HA:calmodulin binding protein) Plekhm1 with TRAFD1-FLAG constructs overexpressed in HEK293 cells. Numbers of constructs in upper panel correspond to lanes in lower panel. Lysates were pulled down with streptavidin-Sepharose followed by western blotting with anti-FLAG (middle blot). The same membrane was probed with anti-HA antibody to monitor pull-down efficiency of Plekhm1 (upper blot). A fraction of the lysate was also probed with anti-FLAG to control for TRAFD1 protein expression (lower blot). Amino acids 37–60 (lane 2 and blue box), containing the zinc-finger, were required for binding to Plekhm1 (lane 2). (B) Reciprocal experiments were done with Plekhm1 constructs diagrammed in the upper panel, with numbers corresponding to lanes in the blots in the lower panel. IP of C-terminally FLAG-tagged full-length TRAFD1 was done with TAP-tagged Plekhm1 constructs. Lysates were immunoprecipitated with anti-FLAG-agarose, blotted, and probed with anti-HA to detect Plekhm1 (middle blot). The same membrane was stripped and probed with monoclonal anti-FLAG antibody to monitor immunoprecipitation efficiency (upper blot). A fraction of the lysate was also probed with anti-HA to control for Plekhm1 protein expression (lower blot). Amino acids 784–986 (of 1059 total), between the second PH domain and the C1 domain, were required for binding TRAFD1 (lane 4 and blue box). The experiments were performed at least 3 times and representative gels are shown. Numbers on left show positions of molecular weight markers. ZF = zinc finger; F = FLAG tag; T = TAP tag; R = RUN domain; * = PH domains; C = C1 domain.

Plekhm1 is a large, modular cytoplasmic protein localized to late endosomes/early lysosomes and involved in vesicle transport and ruffled border formation in osteoclasts. Previously, we and others identified mutations of Plekhm1 that cause osteopetrosis in rats and humans [20] and osteopenia in a human patient [21]. In order to identify interaction partners of Plekhm1, we performed tandem affinity purification and mass spectrometry. Human Plekhm1 fused to the TAP tag [28] was used as bait. TRAFD1 was identified in the resulting set of potential Plekhm1 interaction partners (not shown). To confirm and further analyze the interaction between TRAFD1 and Plekhm1, we performed a series of pull down/immunoprecipitation (IP) experiments. TRAFD1 deletion constructs (**Fig 1A**, lower panel) were tested for their ability to interact with full-length Plekhm1, and the reciprocal experiments were performed with full-length TRAFD1 and deletion constructs of Plekhm1 (**Fig 1B**). Both full-length TRAFD1 and a TRAFD1 N-terminal deletion mutant (aa Δ1–37) bound full-length Plekhm1 (**Fig 1A**). Further truncations of TRAFD1 prevented binding to Plekhm1. The interacting N-terminal region of TRAFD1 is comprised of amino acids 37–60 and is part of the zinc finger domain (28–103 aa). Both full-length Plekhm1 and a more C-terminal region of Plekhm1 (778–986 aa) were immunoprecipitated with FLAG-tagged TRAFD1. Together, these results demonstrated that Plekhm1 and TRAFD1 directly interact and that the zinc finger domain of TRAFD1 is necessary to bind to Plekhm1 between its second PH domain and the C1 domain.

### TRAFD1 mRNA and protein levels increase during osteoclast differentiation

**Fig 2:**
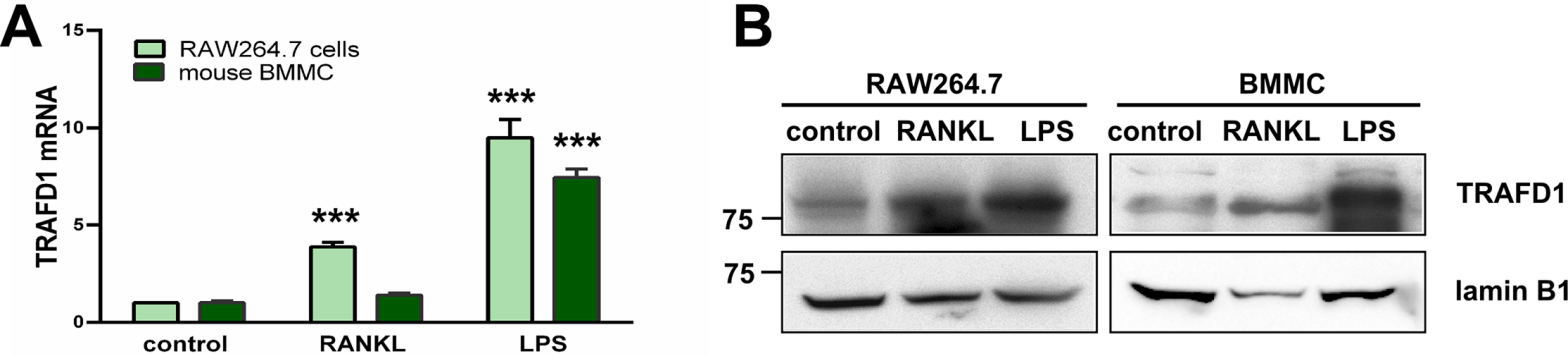
Expression profile of TRAFD1 in mouse cells. (A) Transcript level of *Trafd1* in RAW264.7cells (light green bars) and mouse BMMC (dark green bars) was measured by Q-PCR in cells treated with LPS (100 ng/ml, 7 hours) or RANKL (10 ng/ml RAW264.7 cells, 20 ng/ml BMMC, 3 days). Untreated cells were used as a control. One-way ANOVA was carried out and values are mean + standard deviation (s.d.) of 3 independent experiments. ^***^ *P<*0.0001 *versus* control group. (B) Protein expression of TRAFD1 in RAW264.7 and mouse bone marrow mononuclear cells (BM) treated as in A. Whole cell extracts (50 μg) were analyzed by 8% SDS-PAGE, blotted onto PVDF, and probed with anti-TRAFD1 antibody. Lamin B1 was used as a loading control. The experiments were performed at least 3 times and representative gels are shown.

Previously [24], it was shown that treatment of monocytes with lipopolysaccharide (LPS) increased the level of TRAFD1 protein. To investigate the potential role of TRAFD1 in osteoclasts, we examined its mRNA level in osteoclast cultures. We found that RANKL caused a slight increase in TRAFD1 mRNA in RAW264.7 cells but no change in mouse bone marrow mononuclear cells (BMMC) (**Fig 2A**). Western blot analysis demonstrated that TRAFD1 protein is expressed at a low level in RAW264.7 cells and BMMC, and that RANKL caused increases in TRAFD1 protein in both cell types (**Fig 2B**), suggesting an increase in protein stability, at least in primary cells.

### TRAFD1 co-localizes with Plekhm1 and Rab7 in osteoclasts

**Fig 3:**
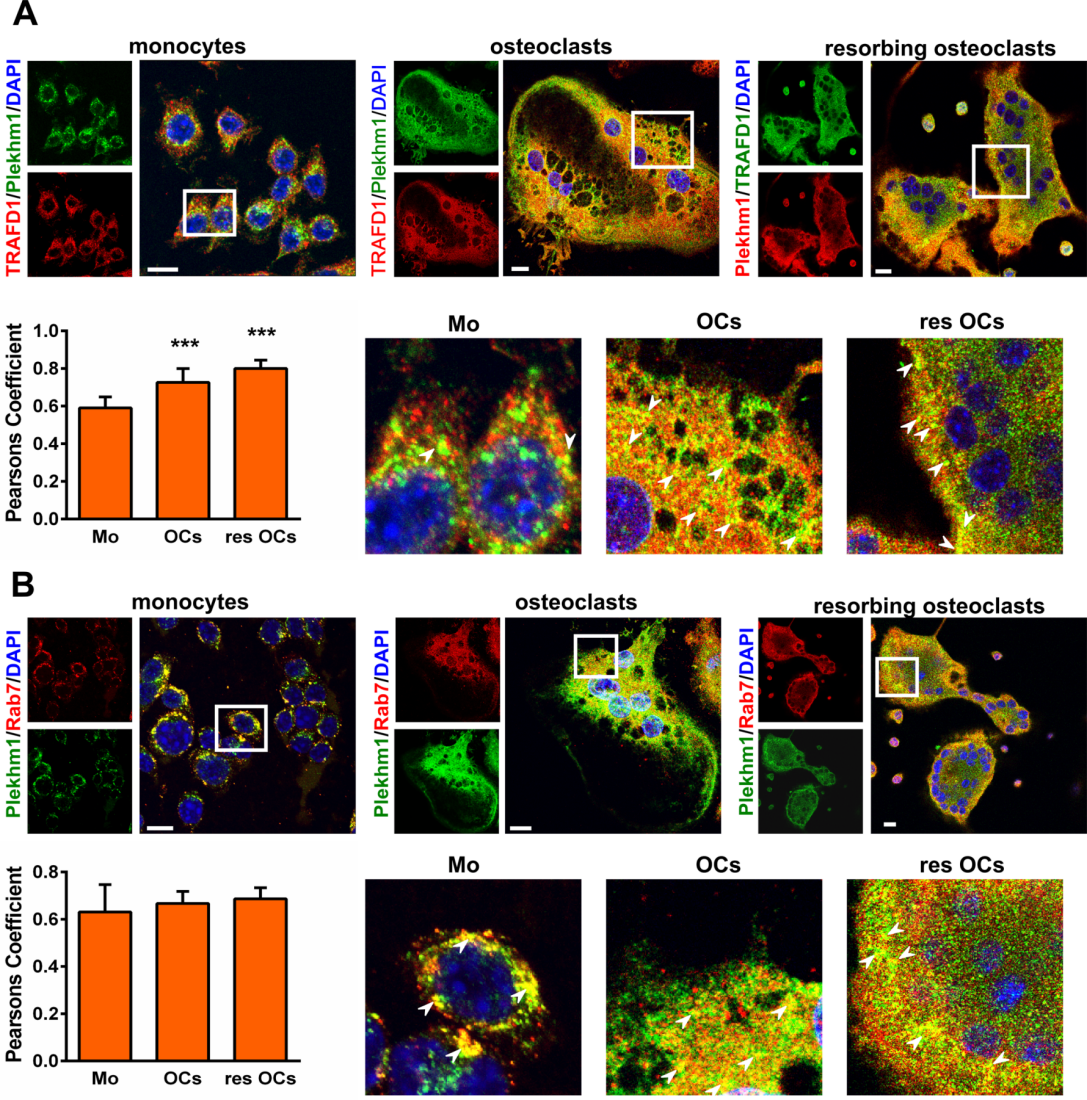
TRAFD1 localization in mouse BMMC. (A) Immunostaining for endogenous TRAFD1 and Plekhm1 was performed on mouse monocytes and on mouse BMMCs treated with RANKL cultured on glass (osteoclasts), or on Osteo Assay plates (resorbing osteoclasts). Confocal microscopy images were obtained using anti-Plekhm1 and anti-TRAFD1 (colors as indicated), and representative images are shown. DAPI staining was used to visualize nuclei. Scale bars = 10 μm. Insets show enlarged regions outlined in white. Arrowheads indicate examples of vesicles where co-localization gives yellow signal. Pearson’s correlation coefficient was used to estimate the co-localization of TRAFD1 with Plekhm1, where 1 represents perfect co-localization and 0 is random co-localization. One-way ANOVA was carried out and values are mean + s.d. of n = 3 independent experiments analyzing at least 4 images/condition. ^***^ *P*<0.0001 *versus* monocytes. (B) Immunostaining for endogenous Plekhm1 and Rab7 and co-localization analysis was performed as in A. Scale bars = 10μm. Values are mean + s.d. of n = 3 independent experiments analyzing at least 4 images/condition. No significant change in co-localization was observed ± RANKL or resorption. M = monocytes; OCs = osteoclasts; res OCs = resorbing osteoclasts.

In order to investigate interactions between TRAFD1 and Plekhm1 in osteoclasts, we analyzed co-localization in BMMC by immunofluorescence. We found an increase in co-localization upon RANKL stimulation (**Fig 3A**). The co-localization further increased when BMMC-derived osteoclasts were actively resorbing on HA plates. Pearson’s correlation analysis showed that co-localization of TRAFD1 and Plekhm1 increased from 0.59±0.05 in BMMC to 0.72±0.07 and 0.80±0.04 in osteoclasts and resorbing osteoclasts, respectively (*P*<0.0001 for monocytes *vs*. osteoclasts and *P*<0.0001 for monocytes *vs*. resorbing osteoclasts). Previously [20], transfection experiments showed that fluorescently tagged Plekhm1 expressed in HEK293 cells co-localized with GTP-bound Rab7, indicating that Plekhm1 localizes to late endosomes/early lysosomes. To assess this in primary osteoclasts, we examined the co-localization of endogenous Plekhm1 with Rab7 (**Fig 3B**). This confirmed that Plekhm1 strongly co-localizes with Rab7 to late-endosomal/early-lysosomal vesicles in mononuclear cells (Pearson’s coefficient = 0.63±0.11) and the level of co-localization does not change in mature osteoclasts (Pearson’s coefficient = 0.66±0.05). Control analysis using 90° rotation of color channel images [34] gave the expected, random value of 0.1±0.05, confirming the significance of the observed co-localizations. The co-localization of endogenous TRAFD1 with Plekhm1 confirms that the protein-protein interactions seen by mass spectrometry and IP assays reflect what occurs in the cell. Further, co-localization of TRAFD1 with Rab7 (Pearson’s coefficient = 0.56±0.11 in monocytes *vs*. 0.71± 0.06 in osteoclasts) places a substantial fraction of TRAFD1 in the same cellular functional compartment as Plekhm1, i.e., late endosomes/early lysosomes of monocytes and differentiated osteoclasts **(S2 Fig)**.

### TRAFD1 impacts resorbing activity of osteoclasts

**Fig 4:**
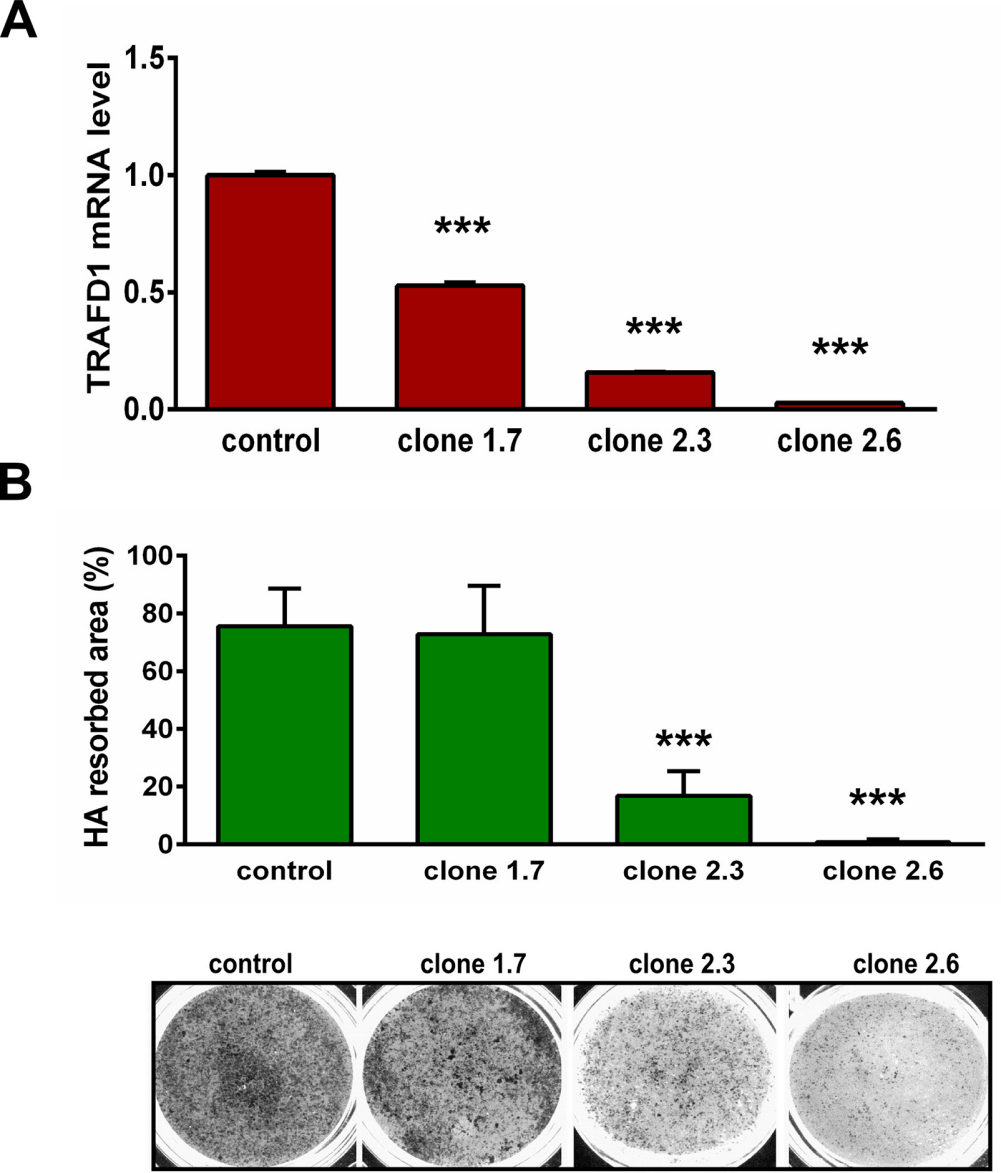
TRAFD1 knockdown inhibits resorbing activity of osteoclasts. (A) Stable knockdown of TRAFD1 was achieved by transduction with lentivirus expressing shRNA (or scrambled control) in RAW264.7 cells. Clones were selected with different degrees of knockdown in the absence of RANKL, as shown (clones 1.7, 2.3; and 2.6). Q-PCR experiments were performed at least 3 times and representative graph shows means+ s.d. of duplicates. ^***^ *P*<0.0001 *versus* control group. (B) Resorption assay was performed with the knockdown clones in A. Cells were cultured on 24-well Osteo Assay (HA) plates for 10 days in the presence of RANKL (10 ng/ml). Cells were removed and the plate was scanned at high resolution. Representative wells are shown on lower panel (black = resorbed area). The percentage of resorbed area over total area of well is indicated on upper panel. Analysis of resorbed area was measured by Image J software. One-way ANOVA was carried out and values are mean +s.d. of n = 3 independent experiments analyzing 3 wells/condition. ^***^ *P*<0.0001 *versus* control group.

In order to investigate potential functions of TRAFD1 in osteoclasts, RAW264.7 cells were transduced with lentivirus encoding shTRAFD1, and stable clones expressing different levels of TRAFD1 mRNA were selected (**Fig 4A**). When the clones were cultured with RANKL on Osteo Assay Plates (hydroxyapatite; HA) for 10 days, defects in resorbing ability were found that reflected the degree of TRAFD1 knockdown (**Fig 4B and 4C**). We used the clone with the lowest TRAFD1 level and the lowest resorption ability, 2.6, designated hereafter as “shTRAFD1,” in subsequent experiments.

**Fig 5:**
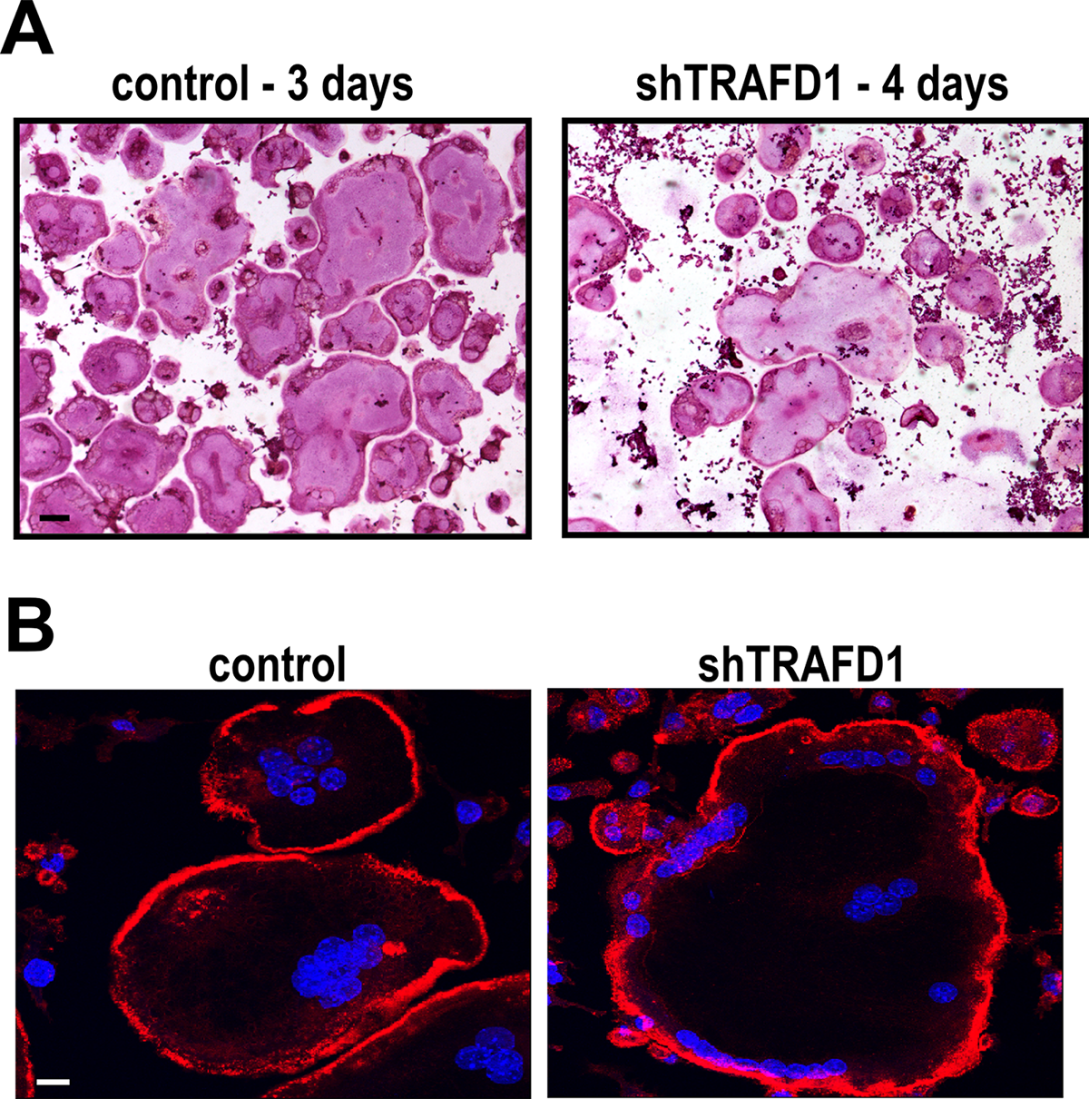
shTRAFD1 cells differentiate in vitro. (A) Cells stably expressing TRAFD1 shRNAs were cultured on 96-well plates in the presence of RANKL (10 ng/ml), fixed, and stained for TRAP on the days indicated. Representative micrographs are shown. Scale bar = 100 μm. (B) Rhodamine phalloidin and DAPI staining of osteoclast-like cells cultured on glass coverslips. shTRAFD1 and control RAW264.7 cells were cultured in the presence of RANKL (10 ng/ml) on 24-well plates with glass coverslips on the bottom. When cells differentiated (3 days for controls, 4 days for shTRAFD1), they were fixed and stained. Representative fluorescence micrographs are shown. Scale bar = 10 μm.

Due to the lack of resorption of HA by shTRAFD1 cells, we investigated their capacity to differentiate and to express factors required for resorptive activity. As shown in **Fig 5**, although they required an additional day (4 days *vs*. 3 days for control cells), shTRAFD1 cells did form multinucleated, TRAP-positive cells (**Fig 5A**). Because we observed a delay in differentiation of shTRAFD1cells, we examined the status of the main osteoclast transcription factor NFATc1. In control cells, we observed increased total NFATc1 protein 24 hours after adding RANKL, and the expression increased until day 3. In shTRAFD1 cells, the NFATc1 level was slightly lower and the translocation of NFATc1 into the nucleus was delayed compare to the control cells, consistent with the delayed differentiation of shTRAFD1 cells (**S3 Fig**).

**Fig 6:**
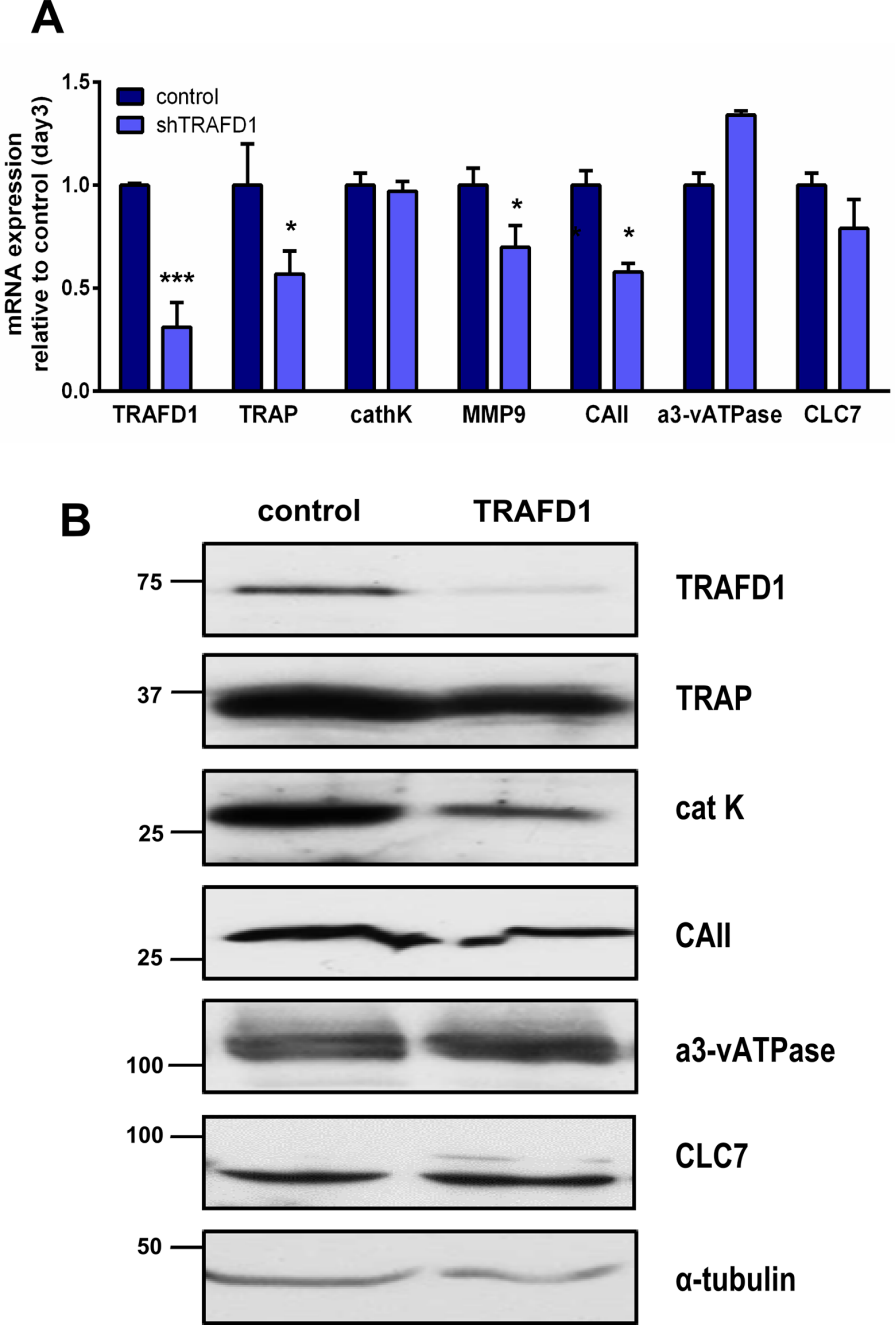
Decreased level of TRAFD1 has minor effects on expression of osteoclast resorption factors. (A) Q-PCR analysis is shown for the indicated genes in differentiating shTRAFD1 cells (4 days in RANKL) *vs*. control cells (3 days in RANKL). The expression of each gene in shTRAFD1 cells is normalized to the expression of control cells on day 3. Student’s *t*-test was carried out and values are mean +s.d. of triplicate determinations in 3 independent experiments. A representative graph is shown. ^*^ *P*<0.05; ^***^ *P*<0.0001 *versus* control. (B) Whole cell protein extracts from differentiated control cells (3 days in RANKL) and differentiated shTRAFD1 cells (4 days in RANKL) were subjected to immunoblotting using the antibodies shown. The experiments were performed at least 3 times and representative gels are shown.

Further, those cells exhibited the actin rings characteristic of osteoclasts, as shown by fluorescent labeling with phalloidin (**Fig 5B**). This prompted us to investigate whether the impaired resorption was the result of reduced levels of known acidification and resorption factors, including the ClC7 chloride channel, cathepsin K, matrix metallopeptidase 9 (MMP-9), carbonic anhydrase II and the osteoclast-specific subunit of the vacuolar proton pump, a3-vATPAse (**Fig 6**). To this end, we measured gene expression levels by Q-PCR and also evaluated western blots of differentiated cells. At the mRNA level, TRAP, MMP-9 and carbonic anhydrase II were significantly reduced (0.57±0.11, 0.7±0.1 and 0.58±0.04 respectively, *P*<0.05) (**Fig 6A**). At the protein level, only cathepsin K showed a reduction. All the other proteins assayed appeared to be present at normal levels. We also observed that differentiation of shTRAFD1 cells with RANKL caused an increase in the level of TRAFD1 mRNA, but we did not observe any changes in the protein level.

**Fig 7:**
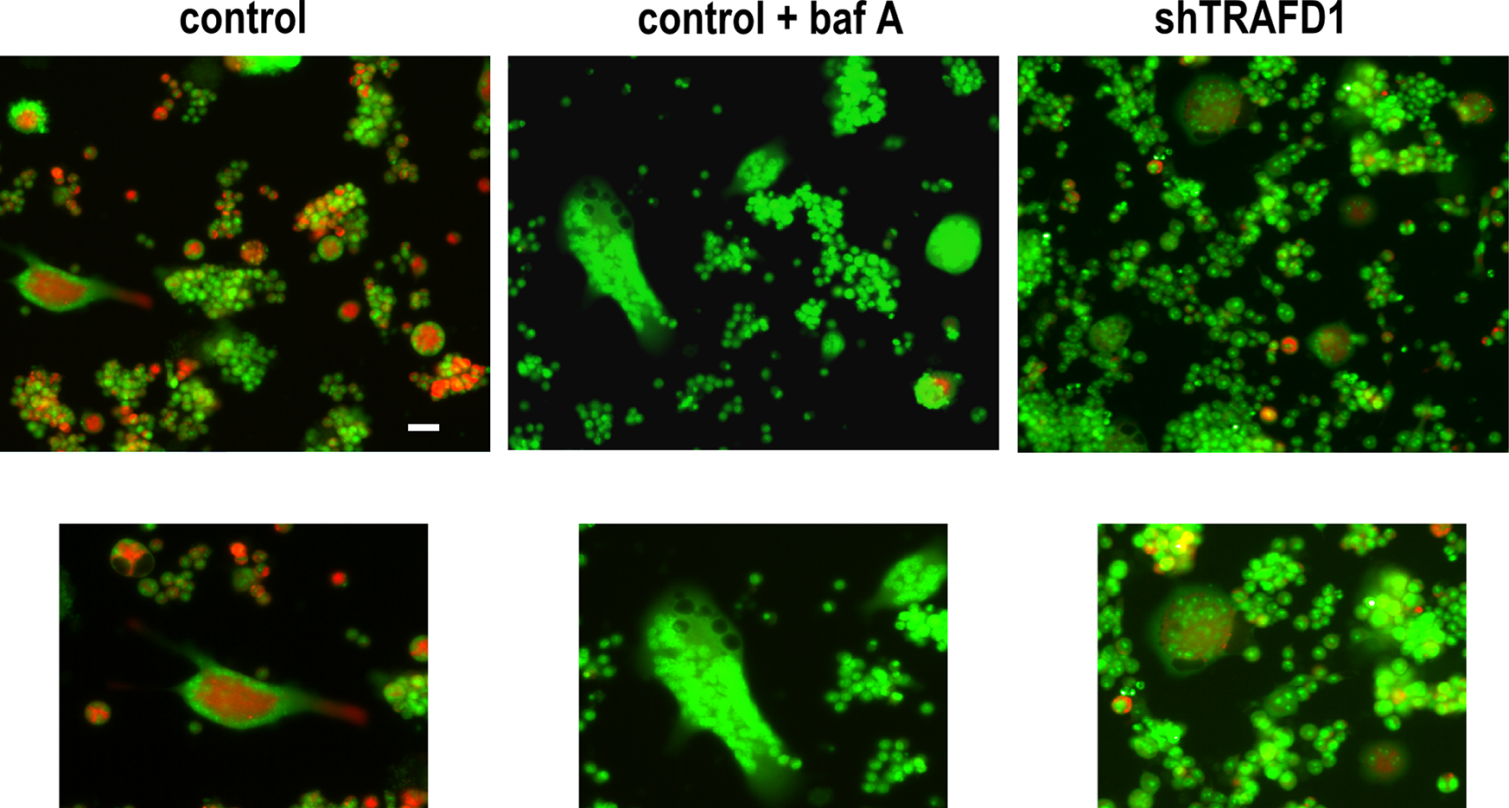
Knockdown of TRAFD1 inhibits acidification of osteoclasts. Acidification assay was performed with shTRAFD1 and control RAW264.7 cells. Cells were cultured on 24-well Osteo Assay plates for 5 days in the presence of RANKL (10 ng/ml) and stained with acridine orange. Representative confocal microscopy images are shown. White rectangles locate areas shown at higher magnification in insets, below. As a control for blocked acidification, some control cells were treated with bafilomycin A (200 nM; control + baf A). Scale bar = 50 μm.

Since actin rings were present in shTRAFD1 cells, and protein levels of resorption factors (with the lone exception of cathepsin K) were normal, we investigated whether acidification was occurring properly in shTRAFD1 cells. Cells were differentiated by culturing with RANKL for 5 days on HA plates, and then the live cells were labeled with the acidotropic probe acridine orange (**Fig 7**). In this assay, osteoclast-like cells with normal acidification display orange-red staining, indicating active production of H^+^ [31,35]. shTRAFD1 cells showed a loss of signal comparable to that seen in wells treated with the proton pump inhibitor, bafilomycin A [36], indicating a role for TRAFD1 extracellular acidification in osteoclasts. Since the proteins required for acidification were present, the lack of acidification may reflect failure of targeting or improper targeting of those factors.

### Knockdown of TRAFD1 impairs TRAP and cathepsin K secretion

**Fig 8:**
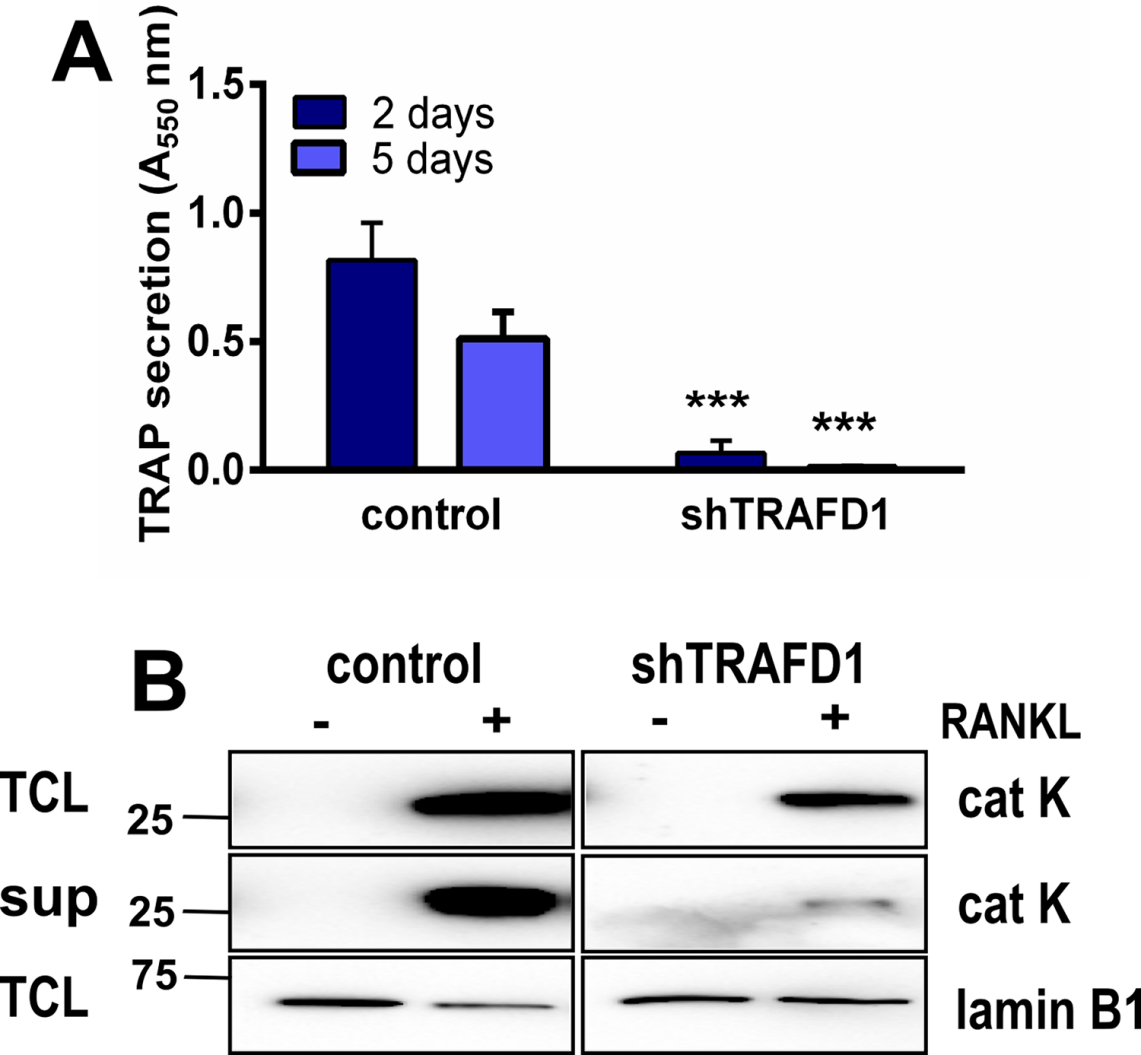
TRAFD1 is required for TRAP and cathepsin K secretion. (A) Differentiated RAW 264.7 cells and shTRAFD1 cells were grown for 5 days on Osteo Assay plates in the presence of RANKL. Conditioned media were collected 2 and 5 days post differentiation and TRAP secretion was measured biochemically. A representative graph is shown. One-way ANOVA was carried out and values are mean +s.d., n = 8 per time point. ^***^ *P*<0.0001 *vs*. controls. (B) TRAFD1 knockdown and control RAW264.7 cells were cultured on Osteo Assay plates in the presence of RANKL. Culture supernatant (sup) was collected 24 hours post differentiation. 40 μl of conditioned media were subjected to western blot analysis for the presence of cathepsin K. Total cell lysates (TCL) from each well served as total protein controls. The same gel was re-probed with lamin B1 antibody. The experiments were performed 3 times and representative gels are shown.

We investigated a possible role for TRAFD1 in osteoclast exocytosis by analysis of TRAP and cathepsin K secretion. TRAP activity was measured biochemically, and cathepsin K was analyzed by western blotting (**Fig 8**). On day 2, TRAP activity in conditioned medium from shTRAFD1 cells was 12-fold lower than in control cell medium (**Fig 8A**). Also, as shown in **Fig 8B**, shTRAFD1 cells secreted markedly less cathepsin K than controls. These data suggest that TRAFD1 is important for targeting cathepsin K and TRAP to their correct location at the ruffled border and are consistent with a role for TRAFD1 in osteoclast vesicle transport and secretion.

### TRAFD1 is associated with vesicles in osteoclasts and its stability is dependent on Plekhm1

**Fig 9:**
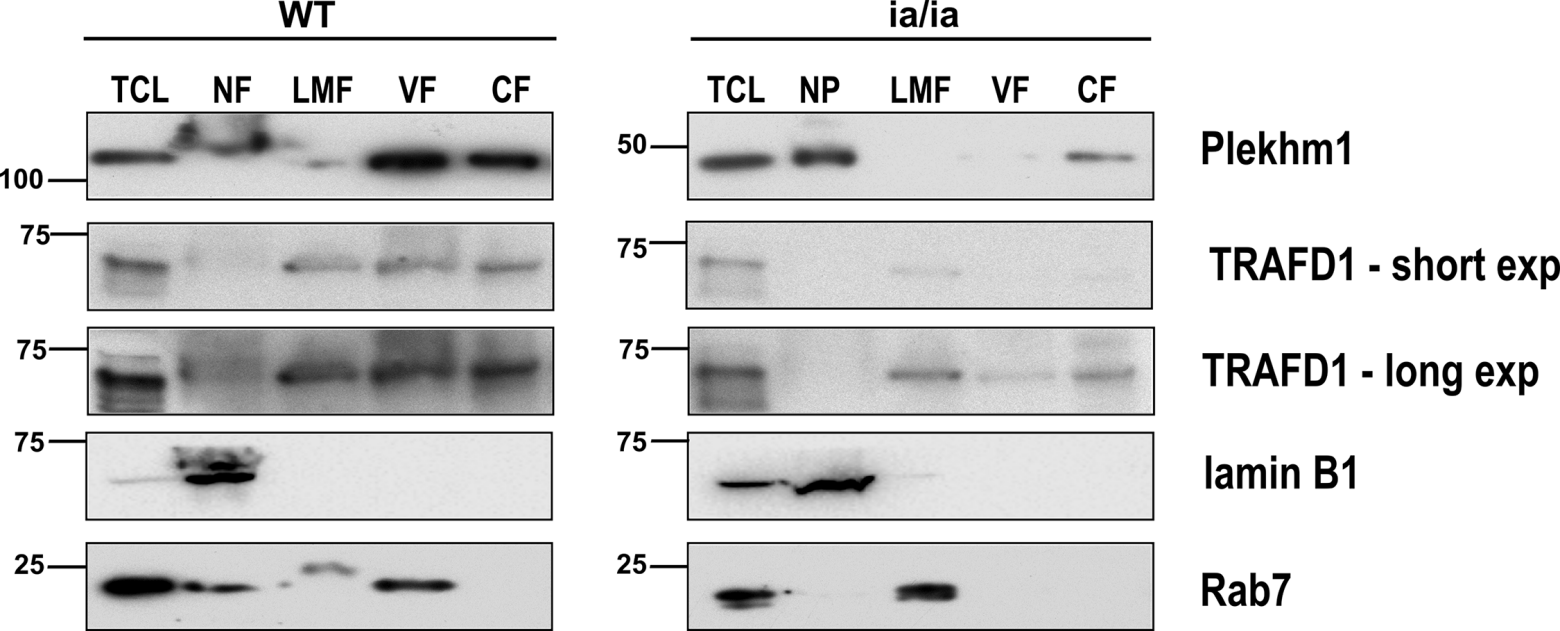
TRAFD1 in osteoclast centrifugal fractionation is Plekhm1 dependent. Osteoclasts from WT and *ia/ia* (Plekhm1 truncation) osteopetrotic rats were osmotically lysed and fractionated by differential centrifugation. All fractions were subjected to western blot. 50 μg of protein from each fraction were loaded per lane. TCL: total cell lysate; NF: nuclear fraction and cell debris; LMF: light mitochondrial and lysosomal fraction; VF: vesicle fraction; CF: cytosolic fraction. The membrane was probed with anti-Plekhm1, anti-TRAFD1, anti-Rab7 (late endosome/early lysosome marker), and anti-lamin B1 (nuclear envelope marker). Truncated Plekhm1 is seen in *ia/ia* rats. The experiments were performed at least twice and representative gels are shown.

To further define the association of TRAFD1 with osteoclast vesicles, we performed sequential ultracentrifugal fractionation of osteoclasts generated from bone marrow cells of WT rats and splenocytes from *ia/ia* rats (which lack bone marrow spaces). Immunoblot analysis of the resulting fractions is shown in **Fig 9**. In WT osteoclasts, most of the Plekhm1 was present in the vesicle fraction (lane VF) and the cytosolic fraction (lane CF). The vesicle fraction in these separations contains endosomes and early lysosomes. Organelle-specific antibody for late endosomes/early lysosomes (Rab7) confirmed the enrichment of those vesicles in this fraction in WT osteoclasts. Unlike Plekhm1, no Rab7 was detected in the cytosolic fraction. TRAFD1 appeared roughly equally partitioned between the light mitochondrial fraction (LMF) lane, the VF lane, and the CF lane. In osteoclasts generated from *ia/ia* rat cells, we observed that their truncated form of Plekhm1 was primarily present mainly in the heavy nuclear pellet (lane NP) and the CF lane, and was absent from the vesicle fraction. This mirrored the altered distribution of Rab7. TRAFD1 in *ia/ia* osteoclasts was detected mostly in the LMF lane; however, with longer exposure we detected a small amount of TRAFD1 in the CF lane and the VF lane. Together, these results suggest that Plekhm1 truncation disrupts both the stability and the vesicular localization of TRAFD1.

## Discussion

TRAFD1, also known as FLN29, was previously linked to signaling by toll-like receptors (TLR) and retinoic acid-inducible gene 1 (RIG-1)-like helicase (RLH), dampening the inflammatory response in macrophages [23,24]. In those studies, TRAFD1 was found to negatively regulate NF-κB signaling by binding to several TRAF proteins, with the TRAF6 interaction the best characterized. In this report, we demonstrate for the first time a specific interaction between Plekhm1 and TRAFD1. Plekhm1 is important in lysosomal trafficking, secretion, ruffled border formation, and bone resorption in osteoclasts [12,20,21]. The results shown here implicate TRAFD1 as also having a role on those processes.

The level of TRAFD1 protein in osteoclast precursors was relatively low, although it increased during RANKL stimulation. We previously observed a similar pattern for Plekhm1 [20]. Confocal microscopy showed that TRAFD1 and Plekhm1 partially co-localize to late endosomes, consistent with our observation of protein-protein interaction in pull-down assays. The co-localization increased upon RANKL stimulation and was even higher in actively resorbing osteoclasts. shRNA knockdown of TRAFD1 in RAW264.7 cells showed that deficiency of TRAFD1 inhibited the resorbing capacity of osteoclast-like cells, but that actin rings formed normally. The knockdown cells were, however, defective in acidification despite the presence of known factors required for that process (CAII, a3-vATPase, ClC7). They were also defective in secretion, with marked reductions in secretion of both TRAP and cathepsin K.

The amino acids of TRAFD1 which bind to Plekhm1, 37–60, are part of a TRAF-like zinc finger domain. That domain interacts directly with amino acids 778–986 (of 1056 total) of Plekhm1. That region of Plekhm1 lies in a conserved sequence between the second of 2 pleckstrin homology (PH) domains and the C1/ZnF domain near the C-terminus. Those are both required for proper interaction with the small GTPase Rab7 [22,37]. Rab7 guides late endosomes/early lysosomes in a GTP-dependent manner, and the GTP-bound state of Rab7 is required for its interaction with Plekhm1 [20]. We also show here that in *ia/ia* osteopetrotic rat cells, which have a truncation at codon 338 of Plekhm1 (of 1059 total), TRAFD1/Rab7/Plekhm1 interactions were disrupted. The distribution of those proteins in sequential centrifugation fractions was altered. In WT osteoclasts, Plekhm1 mainly co-purified with the vesicle fraction, and similar purification was observed for Rab7 and TRAFD1. The truncated *ia/ia* Plekhm1, however, did not co-fractionate with Rab7, and the TRAFD1 level was markedly reduced in the cell lysate and in the vesicle fraction. This suggests that the Plekhm1/Rab7/TRAFD1 interaction is important for the resorbing and secretory activities of osteoclasts and for TRAFD1 stability.

Rab7 and Plekhm1 regulate the secretory lysosomal pathway, which delivers cathepsin K and acidic vesicles to the ruffled border [13]. Plekhm1 is recruited to secretory lysosomes through its interaction with Rab7. Rab7 is known to regulate the fusion between early and late endosomes and between late endosomes and early lysosomes. Also, Rab7 regulates trafficking of late endosomes along microtubules in the minus direction by interaction with RILP and dynein, indicating that Rab7 is also involved in motor protein recruitment [15,38]. Indeed, recent data on LIS1 function in osteoclasts [39] supports that model and adds further evidence linking Plekhm1 to vesicle trafficking. That report showed that LIS1 binds to the dynein/dynactin complex and also interacts with the RUN and PH1 domains of Plekhm1, thus mediating lysosome transport and positioning within the cell. TRAFD1, together with Plekhm1/Rab7/LIS1, may cooperate in the delivery and/or fusion of acidic lysosomes to the ruffled border, thereby facilitating bone resorption.

Interestingly, disruption of dynein-dynactin complexes in resorbing osteoclasts did not impair acidification, but did cause a defect in cathepsin K secretion through the ruffled border [40]. Another study showed that delivery of lysosomes to the ruffled border was blocked when ATP6ap1 (Ac45), an accessory subunit of V-ATPases, was depleted [41]. Those authors demonstrated that Ac45 directly interacts with Rab7 and may act as a bridge between the V-ATPase and Rab7, linked to the transportation of acidic vesicles along microtubules to the ruffled border. Both Plekhm1, by interaction with Rab7, and TRAFD1, by interaction with Plekhm1, are likely to be involved in that process. That model is consistent with our findings, in which we show that osteoclast-like cells depleted of TRAFD1 have acidification factors present, but are unable to acidify vesicles or the resorption lacuna to remove HA. It should be noted that acidic vesicle/vacuole formation does occur in osteoclasts despite the presence of either inactivating (*ia/ia* rat truncation; see **S4 Fig**) or gain-of-function (R714C in an osteopenic patient) mutations in Plekhm1 [21]. The slightly higher pH of the vesicles in the R714C mutant was likely due to an increased rate of vesicle maturation and insufficient time to fully acidify. The very large size of the acidic vesicles in the *ia/ia* rat osteoclasts (**S4 Fig**) suggests blockage of delivery of cargo to the ruffled border, and might explain both the pelleting of Rab7 with the relatively heavy mitochondrial fraction and the lack of ruffled borders in *ia/ia* rat osteoclasts [42]. Together, these observations are consistent with a model in which TRAFD1 is a key to assembly of vesicles having proper membrane insertion and/or activity of the acidification machinery and movement to the ruffled border.

From the results described above, we conclude: 1) TRAFD1 interacts specifically with Plekhm1; 2) that it is required for establishment of effective acidification of the resorption lacuna; 3) that it is significantly associated with Rab7-positive vesicles; and 4) that it is important in the secretory activity of resorbing osteoclasts. The importance of a clearer mechanistic understanding of cathepsin K secretion is underscored by the ongoing development and trials of a cathepsin K inhibitor (odanacatib) to prevent bone loss and tumor metastasis to bone [43]. Cremasco and others [44] demonstrated that the mechanism of secretion of cathepsin K in osteoclasts is PKCδ-dependent and is independent of both ruffled border formation and V-ATPase delivery to the ruffled border. If that dual transport model is correct, we expect that TRAFD1 is involved in both pathways, since both secretion of cathepsin K and TRAP, and acidification were greatly impacted by TRAFD1 deficiency.

## Supporting Information

S1 Fig Co-localization ofTRAFD1 and Plekhm1 in mouse BMMC. Co-localization analysis was done using JACoP plugin for Image J. Pearson’s correlation coefficient values were analyzed using green and red channel of each image **(A)**. As a control and to exclude false-positive results, one of the images were rotated 90°C and the co-localization was measured again **(B).**
(TIF)

S2 Fig Co-localization of TRAFD1 and Rab7 in mouse BMMC. Immunostaining for endogenous TRAFD1 and Rab7 was performed on mouse monocytes and on mouse monocytes treated with RANKL cultured on glass (osteoclasts). Confocal microscopy images were obtained using anti-Rab7 (green) and anti-TRAFD1 (red), and representative images are shown. DAPI staining was used to visualize nuclei. Scale bars = 10 μm. Insets show enlarged regions outlined in white. Arrowheads indicate examples of vesicles where co-localization gives yellow signal. Pearson’s correlation coefficient was used to estimate the co-localization of TRAFD1 with Rab7 (Pearson’s coefficient = 0.56±0.11 in monocytes *vs*. 0.71± 0.06 in osteoclasts). Student *t*-test was carried out and values are mean + s.d. of n = 3 independent experiments analyzing at least 4 images/condition. ^**^ *P*<0.001 *versus* monocytes. Mo = monocytes; OCs = osteoclasts.
(TIF)

S3 Fig Protein expression of NFATc1 in shTRAFD1 cells. **(A)** Western blot analysis of expression levels of NFATc1 in total cell extracts from control and shTRAFD1 cells cultured in the presence of RANKL for 1–3 days. Cells were lysed, blotted, and probed with antibody to NFATc1. α-tubulin was used as a loading control, as indicated. **(B)** Western blot analysis of expression levels of NFATc1 in nuclear and cytosolic fractions of control and shTRAFD1 cells stimulated with RANKL for 1–3 days.
(TIF)

S4 Fig Acidification of *ia/ia* rat osteoclasts. Acidification assay was performed on cells from 7-day-old WT (BMMC) and *ia/ia* (splenocytes) rats. Cells were cultured on 24-well Osteo Assay plates for 5 days in the presence of RANKL (20 ng/ml) and stained with acridine orange. Representative confocal microscopy images are shown. As a control for blocked acidification, some control cells were treated with bafilomycin A (BafA; 200 nM). Scale bar = 50 μm. Virtually all the orange staining in the *ia/ia* cells was in large vesicles, whereas in WT cells, there was staining spread out, presumably under the ruffled borders, which are absent from *ia/ia* rat osteoclasts.
(TIF)

S1 Table Sequence of forward and reverse primers used in Q-PCR. (DOCX)
