# Supplementary figures and images for: TRAFD1 (FLN29) Interacts with Plekhm1 and Regulates Osteoclast Acidification and Resorption
Source: PLoS One. 2015 May 19;10(5):e0127537. doi: 10.1371/journal.pone.0127537 (PMC4438057; doi:10.1371/journal.pone.0127537)

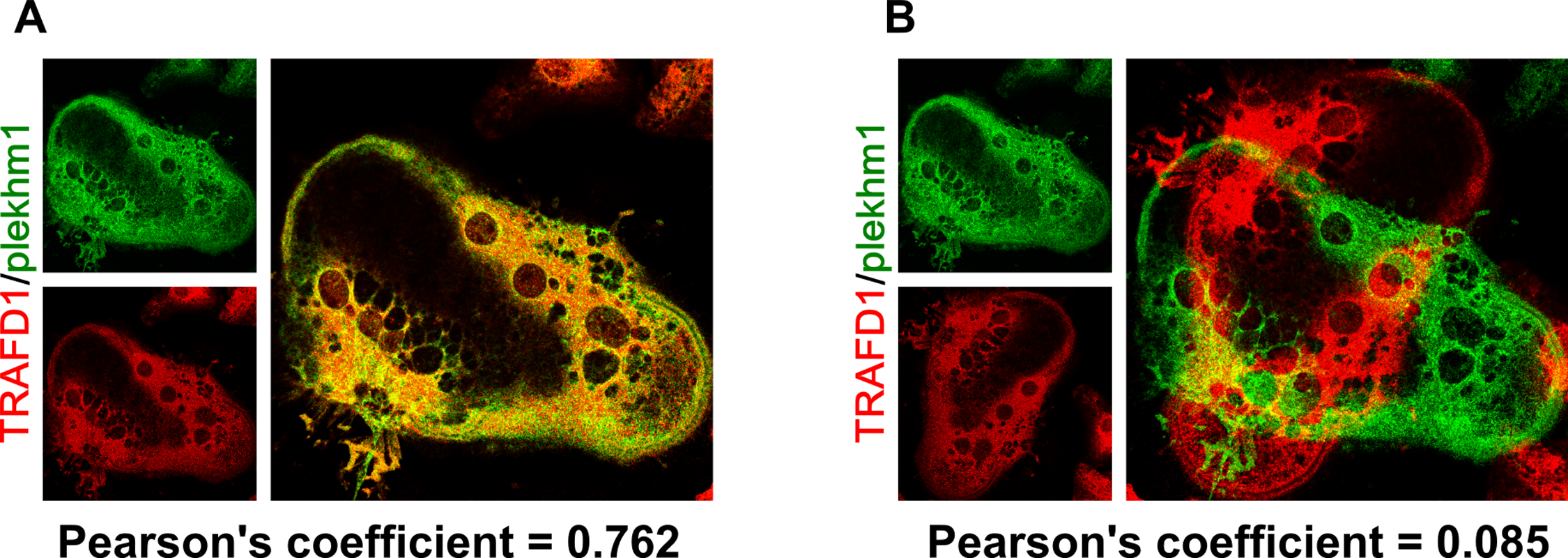

Supplement: S1 Fig — Co-localization analysis was done using JACoP plugin for Image J. Pearson’s correlation coefficient values were analyzed using green and red channel of each image (A). As a control and to exclude false-positive results, one of the images were rotated 90°C and the co-localization was measured again (B). (TIF) [file pone.0127537.s001.tif]

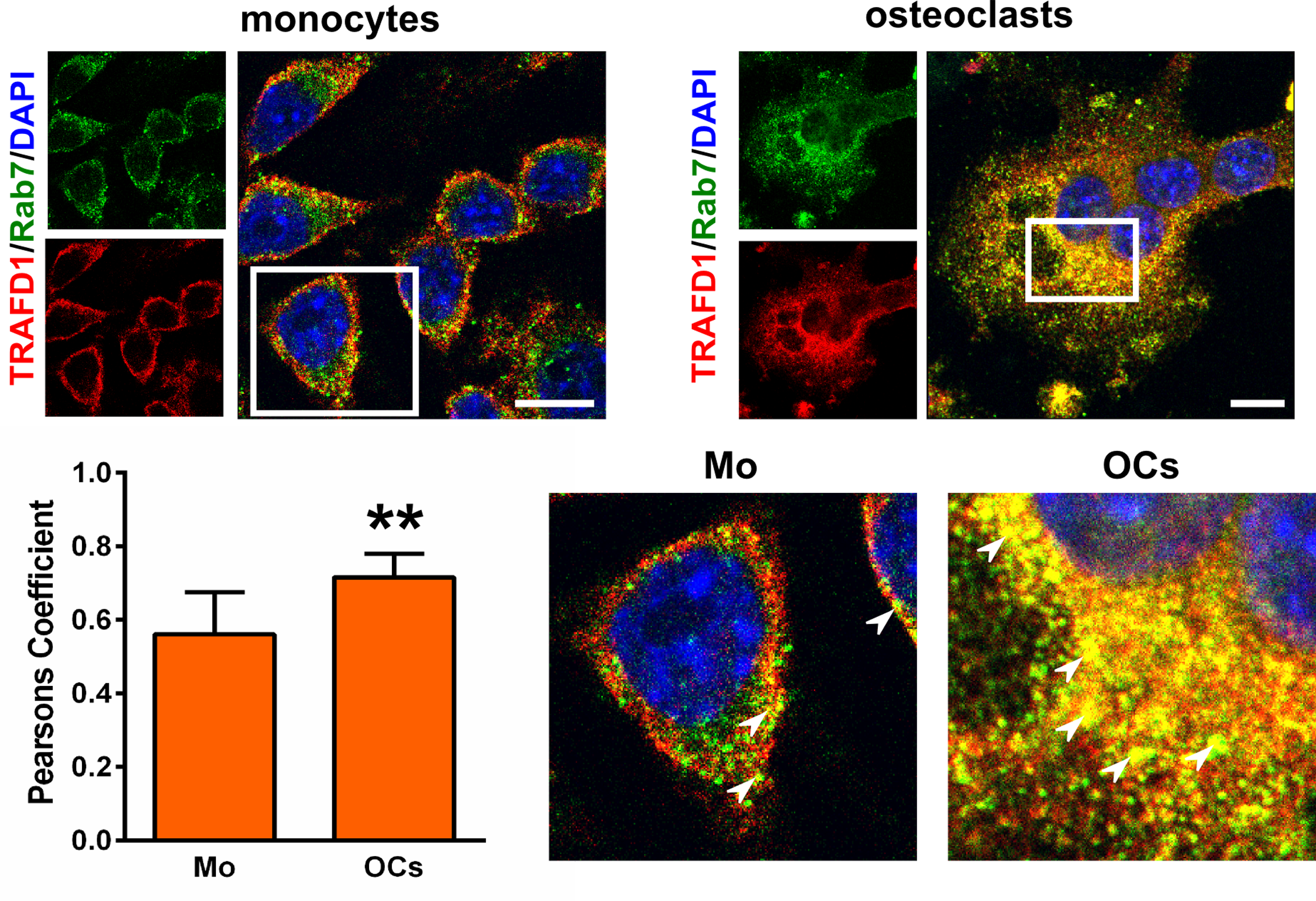

Supplement: S2 Fig — Immunostaining for endogenous TRAFD1 and Rab7 was performed on mouse monocytes and on mouse monocytes treated with RANKL cultured on glass (osteoclasts). Confocal microscopy images were obtained using anti-Rab7 (green) and anti-TRAFD1 (red), and representative images are shown. DAPI staining was used to visualize nuclei. Scale bars = 10 μm. Insets show enlarged regions outlined in white. Arrowheads indicate examples of vesicles where co-localization gives yellow signal. Pearson’s correlation coefficient was used to estimate the co-localization of TRAFD1 with Rab7 (Pearson’s coefficient = 0.56±0.11 in monocytes vs. 0.71± 0.06 in osteoclasts). Student t-test was carried out and values are mean + s.d. of n = 3 independent experiments analyzing at least 4 images/condition. ** P<0.001 versus monocytes. Mo = monocytes; OCs = osteoclasts. (TIF) [file pone.0127537.s002.tif]

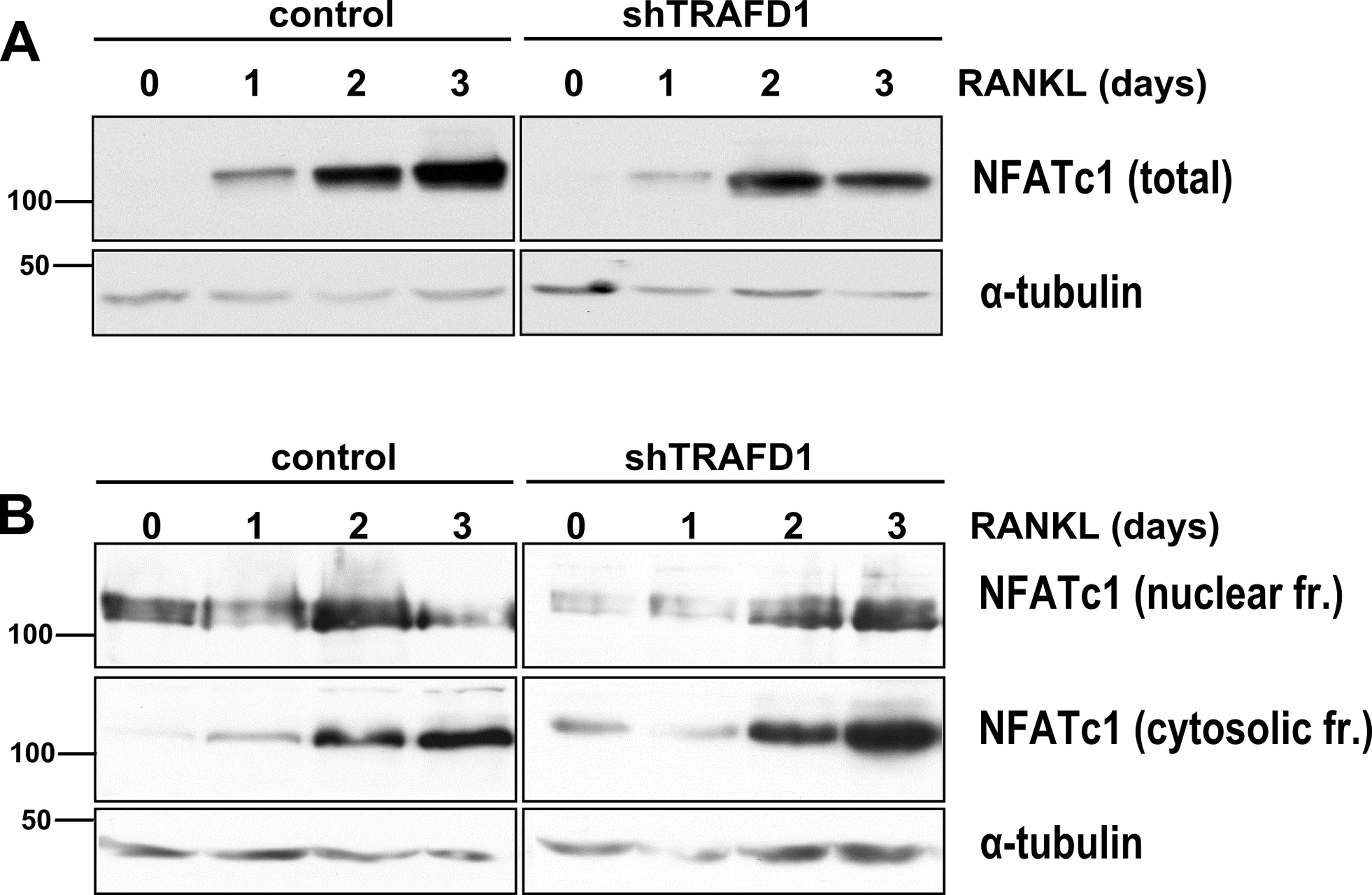

Supplement: S3 Fig — (A) Western blot analysis of expression levels of NFATc1 in total cell extracts from control and shTRAFD1 cells cultured in the presence of RANKL for 1–3 days. Cells were lysed, blotted, and probed with antibody to NFATc1. α-tubulin was used as a loading control, as indicated. (B) Western blot analysis of expression levels of NFATc1 in nuclear and cytosolic fractions of control and shTRAFD1 cells stimulated with RANKL for 1–3 days. (TIF) [file pone.0127537.s003.tif]

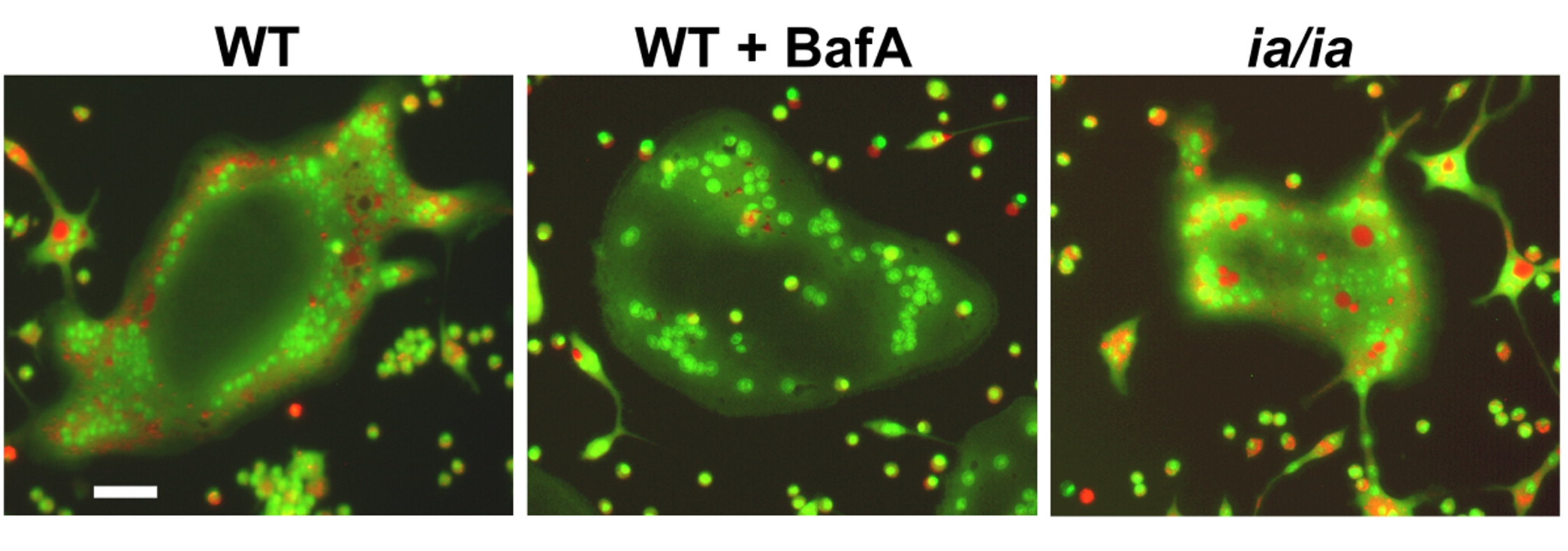

Supplement: S4 Fig — Acidification assay was performed on cells from 7-day-old WT (BMMC) and ia/ia (splenocytes) rats. Cells were cultured on 24-well Osteo Assay plates for 5 days in the presence of RANKL (20 ng/ml) and stained with acridine orange. Representative confocal microscopy images are shown. As a control for blocked acidification, some control cells were treated with bafilomycin A (BafA; 200 nM). Scale bar = 50 μm. Virtually all the orange staining in the ia/ia cells was in large vesicles, whereas in WT cells, there was staining spread out, presumably under the ruffled borders, which are absent from ia/ia rat osteoclasts. (TIF) [file pone.0127537.s004.tif]
