# Supplementary material for: TRAFD1 (FLN29) Interacts with Plekhm1 and Regulates Osteoclast Acidification and Resorption
Source: PLoS One. 2015 May 19;10(5):e0127537. doi: 10.1371/journal.pone.0127537 (PMC4438057; doi:10.1371/journal.pone.0127537)
Supplement: S1 Table — (DOCX) [file pone.0127537.s005.docx]

**S1 TABLE.**

| **Gene** | **Primer sequence** | **Accesion no.** |
| --- | --- | --- |
| *Acp5* | Forward 5’-CCAGCGACAAGAGGTTCC-3’  Reverse 5’- AGAGACGTTGCCAAGGTAT-3’ | NM_001102405 |
| *Atp6v0d2* | Forward 5’-AAGCCTTTGTTTGACGCTGT-3’  Reverse 5’-GCCAGCACATTCATCTGTACC-3’ | NC_000070 |
| *Ctsk* | Forward 5’- GATGAAATCTCTCGGCGTTT -3’  Reverse 5’-CACTGGTCATGTCTCCCAAG-3’ | NM_007802 |
| *Car2* | Forward 5’- CTCAGGGAGCCCATTACTGT -3’  Reverse 5’- TCCTCATTGAAGTTCAGCGT -3’ | NM_009801 |
| *Clcn7* | Forward 5’- CTGAGAAGAGCGTTGTCAGC -3’  Reverse 5’-TTCCATGACCCACCAGGCTCC -3’ | NM_011930 |
| *Dcstamp* | Forward 5’-AAGCTCCTTGAGAAACGATCA-3’  Reverse 5’-CAGGACTGGAAACCAGAAATG-3’ | NM_029422.4 |
| *Mmp9* | Forward 5’- ACGACATAGACGGCATCCA-3’  Reverse 5’-GCTGTGGTTCAGTTGTGGTG-3’ | NM_013599 |
| *Ocstamp* | Forward 5′-TGGGCCTCCATATGACCTCGAGTAG-3′;  Reverse: 5′- CAAAGGCTTGTAAATTGGAGGAGT-3′ | NM_029021 |
| *Plekhm1* | Forward 5’- GAAGCTTGTGGTTTCCTCTCC-3’  Reverse 5’-GAGACCTGCAGCGCTCTTTA-3’ | NM_183034 |
| *Rplp0* | Forward 5’-TGTTTGACAACGGCAGCATTT-3’  Reverse 5’-CCGAGGCAACAGTTGGGTA-3’ | NM_007475 |
| *Tcirg1* | Forward 5’-TCAGGCTGGAGTGACGAGTA -3’  Reverse 5’-GGTCCAAGGAAGACACCAGT-3’ | NM_016921 |
| *Trafd1* | Forward 5’- GCCCACATCTCTAGGTGACATAA -3’  Reverse 5’-GGGTACAGCTCCTCACAGAACT-3’ | NM_172275 |
